# A feasibility study to prevent falls in older people who are sight impaired: the VIP2UK randomised controlled trial

**DOI:** 10.1186/s13063-016-1565-0

**Published:** 2016-09-26

**Authors:** Heather Waterman, Claire Ballinger, Caroline Brundle, Sebastien Chastin, Heather Gage, Robert Harper, David Henson, Bob Laventure, Lisa McEvoy, Mark Pilling, Nicky Olleveant, Dawn A Skelton, Penelope Stanford, Chris Todd

**Affiliations:** 1 University of Cardiff, Eastgate House, 35-43 Newport Road, Cardiff, CF24 0AB UK; 2 University of Southampton, Southampton, UK; 3 Bradford Teaching Hospitals NHS Foundation Trust, Bradford, UK; 4 Glasgow Caledonian University, Glasgow, UK; 5 University of Surrey, Surrey, UK; 6 Central Manchester University Hospitals NHS Foundation Trust, Manchester, UK; 7 Manchester Academic Health Sciences Centre, Manchester, UK; 8 University of Manchester, Manchester, UK; 9 University of Loughborough, Loughborough, UK; 10 Trafford General Hospital, Trafford, UK

**Keywords:** Older people, Visual impairment, Sight loss, Exercise, Home safety, Falls

## Abstract

**Background:**

Published evaluations of successful interventions to prevent falls in people with sight impairment (SI) are limited. The aim of this feasibility study is to optimise the design and investigation of home safety (HS) and home exercise (HE) programmes to prevent falls in older people with SI.

**Methods:**

A community-based feasibility study in north-west England comprising a three-arm randomised controlled trial (RCT) allocated participants to (1) a control group receiving usual care and social visits, (2) an experimental group receiving the HS programme and (3) an experimental group receiving the HS + HE programme. Participants were community-dwelling, aged 65 years and older and sight impaired. Primary outcome data on falls were collected continuously over 6 months. Secondary outcomes on physical activity (self-report and instrumented) and adherence were collected at baseline and 3 and 6 months for HE and at 6 months for the HS programme. Costs for the HS and HS + HE groups were calculated from logs of time spent on home visits, telephone calls and travel. The research assistant and statistician were blinded to group allocation.

**Results:**

Altogether, 49 people were recruited over a 9-month period (randomised: 16 to control, 16 to HS, 17 to HS + HE). The interventions were implemented over 6 months by an occupational therapist at a cost per person (pounds sterling, 2011) of £249 (HS) and £674 (HS + HE). Eighty-eight percent (43/49) completed the trial and 6-month follow-up. At 6-month follow-up, 100 % reported partially or completely adhering to HS recommendations but evidence for adherence to HE was equivocal. Although self-reported physical activity increased, instrumented monitoring showed a decrease in walking activity. There were no statistically significant differences in falls between the groups; however, the study was not powered to detect a difference.

**Conclusion:**

It is feasible and acceptable for an occupational therapist to deliver HS and HE falls prevention programmes to people with SI living independently in the community. Future studies could access Local Authority Registers of people with SI to improve recruitment rates. Further research is required to identify how to improve adherence to HE and to measure changes in physical activity before conducting a definitive RCT.

**Trial registration:**

ISRCTN53433311, registered on 8 May 2014.

## Background

Older people with sight impairment (SI) may have several risk factors for falls including impaired balance, muscle weakness as well as poor visual contrast sensitivity and acuity, reduced visual field and decreased depth perception [1, 2]. Falls in the older population can lead to serious health and social consequences including hospitalisation, permanent disability, change of residence, loss of independence, isolation and depression [3–5]. Preventing the sequelae of falls in community-dwelling older people is an effective health-promotion strategy [6, 7].

The most recent Cochrane systematic review reports that multicomponent home-based exercise (HE) programmes and home safety (HS) assessment and modification programmes reduce the rate of falls and the risk of falling in older people who live in the community [8]. In a randomised controlled trial (RCT) carried out in New Zealand with older people with SI, HS modifications delivered by an occupational therapist (OT) resulted in a significant reduction in the risk of falling (incident rate ratio (IRR) 0.59, 95 % CI 0.42–0.83), but the group receiving HE showed a nonsignificant increase in the risk of falls (IRR 1.15, 95 % CI 0.82–1.61) [9]. It seems that people who received both HS and HE interventions may have received conflicting messages from the OTs and physiotherapists who delivered them which may have detrimentally affected adherence to interventions and the outcome of the study as those who received only HS (IRR 0.39, 95 % CI 0.24–0.62) or only HE (IRR 0.79, 95 % CI 0.48–1.28) in subanalyses suggested that the programmes alone may be more efficacious [9]. The HE intervention primary analysis was evaluated on an intention-to-treat basis, but when analysed per protocol those participants who adhered to the HE protocol had significantly fewer falls than those who did not [9]. In another smaller study, there was a trend for fewer falls but no significant difference in the rate of falls in people with SI aged 50 years and older between those who received 12 lessons on the Alexander technique and those who did not [10]. Adherence to HE was good as an instructor visited participants weekly at home. Peer mentors (PMs), defined as peers who provide encouragement to help adherence to exercise, have also been used successfully with older people without SI [11].

As recommended by the MRC framework and guidance on developing and evaluating complex interventions [12, 13], we aimed to carry out a feasibility study [14, 15] based on the New Zealand study of HS and HE interventions for preventing falls and falls-related injuries in community-dwelling older people with SI in north-west England prior to a subsequent clinical trial. We designed our feasibility study according to the National Institute for Health Research, UK, (NIHR), definition as research carried out before a main study that aims to gather information on parameters (see objectives below) which are useful in the design of the main study [14, 15]. It was comprised of a small RCT with a sample size that was adequate to estimate the important parameters for the main study [14, 15] and so was in keeping with the purpose of a feasibility study. In order to ensure that the programmes were suitable for people with SI, we undertook qualitative work to identify how best to adapt HS and HE interventions to maximise adherence [16]. Specific objectives of the current feasibility study were:

- To determine the willingness of clinicians to identify and introduce the study to potential participants
- To determine the rate of recruitment and attrition, and willingness of patients to be randomised
- To monitor the response rate to follow-up assessments of primary and secondary outcome measures
- To estimate the variability of outcome measures and statistical parameters needed to calculate sample size for a definitive trial
- To investigate adherence rates to the HS modifications and HE programmes
- To assess the resource implications and costs of the interventions, and conduct a preliminary cost-effectiveness analysis
- To assess the feasibility of HE and HS data collection and analysis
- To explore the acceptability of the interventions from the participants’ perspectives

## Methods

### Study design and ethics

**Fig. 1:**
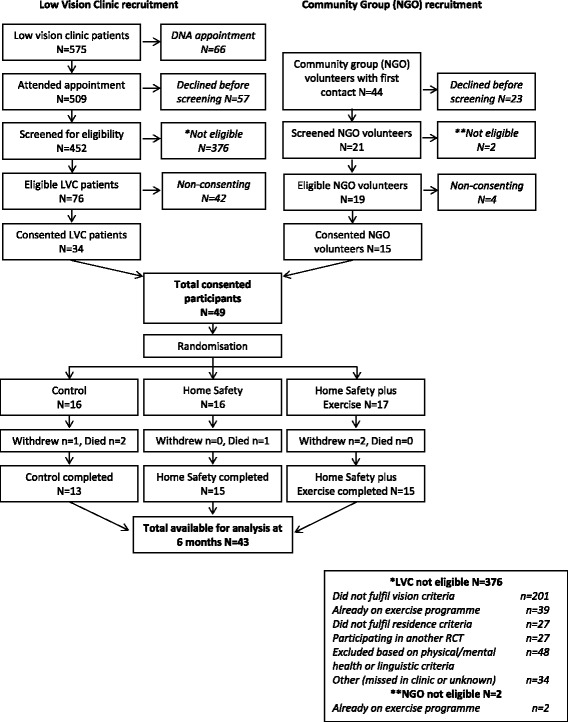
Consolidated Standards of Reporting Trials (CONSORT) diagram of recruitment and flow of participants through the trial

This feasibility study was a three-arm RCT (Fig. 1). Participants in the control group received usual care plus social visits, whilst those in experimental group 1 received the HS programme and experimental group 2 received the same HS intervention plus the HE programme.

### Sample, sample size, recruitment, consent and randomisation

Patients were included in the study if they matched vision-related criteria and were aged 65 years and over, independently living in the community, able to walk around their own residence, cognitively able to participate and able to understand the study requirements. They were excluded if they were already receiving an OT or physiotherapist intervention or home safety assessment and modification or exercise intervention including attendance at a Falls Clinic or did not achieve between 7 and 10 on the Abbreviated Mental Test [17]. Vision-related inclusion criteria were:

1. Binocular visual acuity >0.6 LogMAR (Snellen equivalent 6/24), and/or
2. Moderate visual field loss, defined as affecting more than 20 % of the test locations used in a binocular Esterman test

Vision was measured by LogMAR letter charts, contrast sensitivity (Pelli-Robson chart) and a perimetry test (Esterman test).

The intended sample size was 30 participants in each group (a simple randomisation 1:1:1 ratio) allowing for 10 % attrition [18] sufficient to estimate the percentage of people who fall and the rate of falling within 6 months for each group (our primary outcomes), thus facilitating a power calculation for a future definitive trial.

A process evaluation was conducted in order to understand the feasibility of the intervention and to improve the design of the main study. Hence, all participants who completed the study were included in a qualitative exploration of the acceptability of the interventions including the control group, who were asked about the social visit.

Participants were initially identified from a low vision clinic by NIHR research staff at a hospital in north-west England. Due to slow recruitment, the research assistant sought additional volunteers from meetings with vision-related charities. Informed written consent was obtained in all cases. Baseline data collected by the research assistant at either the hospital clinic or participants’ homes included: age, sex, visual acuity, visual fields, ethnicity, social class and falls history. Participants were then independently randomised by the Clinical Trials Unit via a web-based secure randomisation service which informed the OT or lay visitor coordinator of the group assignment. For pragmatic reasons each participant received the intervention to which they were randomised for 6 months, long enough to assess the feasibility of both interventions rather than providing an optimal dose of exercise. Interventions were commenced within 2 weeks of randomisation. The research assistant and statistician were blinded to group allocation but clearly patients, the OT delivering the intervention, social visitors and PMs could not be blinded to group allocation. However, specific hypotheses being tested were not disclosed. The study was planned with the safety of participants uppermost in our minds. We were observant for how we could prevent falls as unintended consequences of our interventions. These are discussed below in turn in the description of the interventions for each arm of the study.

### Interventions

#### Group 1: usual care plus social visits (control group)

Those allocated to the control group received usual care from the NHS, but in addition received three social visits and two telephone calls by lay visitors (volunteer student nurses, alumni and members of staff from the university). Social visits were to control for the possibility that social contact may reduce falls or influence lifestyle or quality of life variables and were on the same schedule as PMs. Lay visitors had Criminal Record Disclosure and Barring checks and received travel expenses and up to the value of £40 in shopping vouchers depending on how many visits were made. Each lay visitor had a 2-h training session which included visual impairment awareness, a discussion of the study protocol and safety/lone worker issues. The social visitors discussed general topics including holidays, the weather, current affairs, and provided no clinical advice. We did not anticipate any new safety concerns for the participants arising out of this arm of the study but should the need have arisen they were told to suggest that the participant talk with their general practitioner about any concerns.

#### Group 2: home safety intervention only (HS group)

An experienced specifically trained OT used an amended version of the Westmead Home Safety Assessment for those with SI to discuss with participants the physical and environmental hazards present in their homes [19, 20]. This resulted in a jointly agreed action plan incorporating participant needs and views. The action plan focussed on how the participant could alter their environment to reduce the likelihood of falls. The OT attended the initial assessment of the participant’s home prepared to make any smaller safety modifications including replacing light bulbs, adding non-slip matting, and placing high-contrast orange tape on hazardous edges/steps. This was instead of asking the person to make the changes themselves as they may have found it difficult to do or it may have been unsafe for them to have done so. Larger, more complicated home modifications, for example, installation of grab rail, were requested from the NHS. The attendant OT was already working in the NHS and obtained referrer status from all the boroughs to be able to order equipment or adaptations. As in the original New Zealand study, the underpinning approach to promote therapist-client collaboration was the Canadian Model of Occupational Performance [21]. A comprehensive assessment and intervention identifies the full range of potential hazards and raises awareness about the environment, how to negotiate it and how to solve problems [21]. Support for and monitoring of follow-up is also a crucial element of the intervention [19]. The OT visited twice and contacted the participant by telephone at 6 months to find out whether each recommended modification had been completely, partially or not carried out.

#### Group 3: home exercise programme and home safety intervention (HS + HE group)

As well as the HS intervention described above, participants in this group received a shortened version of the Otago Exercise Programme (OEP) to perform over 6 months [22]. The exercises, stressing both strength and balance, are individually prescribed, progress in difficulty, and are undertaken for 30 min at least three times per week. A walking plan was also agreed with all participants to be undertaken at least twice per week. Amendments to the OEP were made so it could be delivered safely to people with SI: (1) the OT used alternative media to explain exercises (including audio), (2) the exercise documentation was presented in large font with black letters on a yellow background as recommended by the Royal National Institute for the Blind, (3) ankle weights used to enable progression of exercises were selected that people with SI could easily use them, and (4) touch was used to demonstrate rather than pictures. Then, in the process of negotiating the exercises with the participant, the OT jointly planned the exercises, was sensitive to the participant’s self-esteem, was careful to build exercise into everyday activities, explained benefits in positive terms (e.g. independence, safety, confidence), set short-term goals, identified and reduced exercise hazards and encouraged attempts at exercise. Participants were advised to exercise three times per week for 30 min and increase the duration of walking over time, first indoors then outdoors if willing. It was explained that they should find the exercise level challenging and were encouraged over time to reduce their support and increase the ankle weights. The OT was supported by 12 volunteer PMs, aged over 60 years, whose role was to encourage participant adherence to exercise but not to deliver HE; they played no direct role in the HS intervention. The OT visited twice, and a PM visited three times and rang twice over the 6-month period, to encourage the person to adhere to the exercise programme.

### Training and supervision of the OT and PMs

Training and continued oversight of the HS and HE programmes and support of the OT and PMs was provided by experienced OEP and Westmead trainers (DAS and CB). The OT was trained to introduce the HE programme to patients at their HS assessment.

Volunteer PMs were aged 60 years or older and lived near the participants and were trained using the Senior Peer Mentor Physical Activity Programme for Older People [23]. Volunteers were recruited through word of mouth and from the University of the Third Age (U3A) in Manchester [24]. Before they agreed to take part they were informed about the study and the inclusion criteria. Inclusion/exclusion criteria for PMs: PMs needed to be physically active, cognitively intact, willing to have a criminal record check as they would be working with vulnerable older people, and willing to commit to the study for 6 months. Seventeen mentors were recruited, 16 attended training following which four withdrew explaining, for example, that they did not have the time. A 2-day training course was run by members of the research team (RMEL and DAS). The training included specific guidelines on home visits to aid adherence and also how to join the participants in the exercises (PMs did not instruct on the actual exercises nor offer advice on HS modifications). PMs were indemnified by the sponsor organisation. PMs received a fee for attending meetings and training days and could claim for travel expenses [25]. Each mentor was assigned one or two participants.

### The acceptability of the interventions

The research assistant interviewed participants in their homes after they completed the trial to explore the acceptability of the interventions. The interviews were digitally recorded and questions were mostly open-ended. Those in the intervention groups were asked about the HS and HE, where appropriate, specifically on whether they found them acceptable, understood the purpose and whether and why they participated or not. The control group were asked about their experiences of the social visits.

### Outcome measures

The primary outcome measures were falls and injurious falls defined according to ProFaNE group definitions [26–28] and were collected prospectively over the intervention and follow-up periods. As in other studies, a falls calendar comprising a single postcard for each month (prepaid business reply) requested the participant to indicate whether or not they had had a fall on each day of the month [28]. Any slips or near falls were also recorded. Our previous experience showed that most older people with SI will complete and return the forms as requested. Participants who did not complete the calendar or who reported falling were contacted each month by telephone. Data collection on falls commenced immediately after randomisation and ceased at 6 months after allocation of each participant.

Secondary outcomes on adherence rates to the HS recommendations were measured at 6 months through a telephone call by the OT to the participant to enquire about completion of agreed safety modifications. Full adherence was defined as when the participants had completed all modifications and partial as when it was less than complete. Self-report and instrumented measures of adherence to undertaking the prescribed exercises and walking programme were assessed at baseline, 3 months and 6 months using exercise calendars (HS + HE only), a telephone questionnaire for self-reporting physical activity (Phone-FITT) [29] and instrumented monitoring of physical activity using body fixed sensors (activPAL sensor; http://www.paltechnologies.com/) [30]. The activPAL is a small device worn on the thigh and validated for use with older adults [31] and reliably detects the start and duration of periods of sedentary behaviour (sitting and lying), standing (upright) and walking time and steps taken. Data from the activPAL monitors were downloaded and processed using PALtechnology software. Data were then analysed using MATLAB to obtain average daily statistics for time spent sedentary (lying and sitting), upright (walking and standing), walking and the number of steps taken over 24-h periods.

To assess methods of collecting ‘quality of life’ and psychosocial variables, we piloted reliable and valid questionnaires: quality of life measure (SF-12) [32], visual disability (VCM1) [33], attitudes and beliefs towards falls-related interventions (AFRIS) [34], ‘fear of falling’ falls self-efficacy (Short FES-I) [35, 36]. Each scale was administered at baseline and 6 months.

### Analysis

Analysis was on an intention-to-treat basis and was largely descriptive using SPSS 20 [37]. In order to inform the main study, analysis concentrated on estimating (1) the overall recruitment rate and the attrition rate per arm [18, 38], (2) the percentage of participants who had adhered to the safety modifications and exercise programme, and (3) the percentage of people who fall and the fall rate for each arm. Those in the control group were used as part of the sample size calculations for the main study. Differences between study arms in the percentage of people who fall and the fall rates with exact confidence intervals for Poisson rates were estimated using StatsDirect [39]. Differences in fall rates between groups were tested using a negative binomial model which included time (calendar days) as a covariate. These were interpreted cautiously as the feasibility study was not powered to detect statistically significant differences. General linear models for repeated measures using time as a covariate, with group and group-by-time interaction terms as explanatory variables were applied, to test for overall group differences and support a descriptive interpretation of results for Phone-FITT FDI (Frequency Duration Intensity), and activPAL sedentary time, upright time, walking time and step count measures. The aim of the qualitative data analysis was to illuminate the quantitative findings, specifically focussing on the acceptability of the interventions. Qualitative data were transcribed and anonymised, and then coded and themes grouped into categories using Framework Analysis [40, 41] implemented using Nvivo version 9 [42].

### Economic analysis

To estimate the costs of the programmes, human resources used in delivering the interventions (home visits and telephone calls by the OT in HS group, and the OT and PMs in the HS + HE group) were collected from staff time and travel records. Nationally validated unit costs were applied to time reported by OTs and PMs [43]. A distinction was drawn between patient contact time (home visits and telephone calls) and patient-related, but non-patient-facing time (travel and writing notes or letters). Rates for family support workers were used to infer a value for PM time [43]. Travel costs (based on local NHS reimbursement rates) were added to human resource costs to gain a total cost at the individual patient level from which the average costs per participant in each group were calculated. Home safety recommendations and actions were analysed descriptively. Expenditures by participants or other agencies to implement modifications were not costed. It was intended that costs were considered in relation to the primary outcome (falls prevented) in a preliminary analysis of cost-effectiveness.

## Results

### Participant recruitment

All clinicians were willing to approach potential participants, but locating enough participants who matched the inclusion criteria from low vision clinics proved difficult. Thus, participants were also recruited from vision-related charities. The CONSORT diagram demonstrates that eligible participants were willing to be recruited. Forty-nine participants were recruited and randomised from March to October 2012 (Fig. 1) and 43 (87.8 %) completed the study (three died, three withdrew). There were no serious adverse events that could have been attributed to the interventions of the study. The range of ages of participants was 65–96 years, mean 81.4 years (SD 7.6) and the groups did not differ in age (Table 1). The demographic characteristics of groups were generally comparable and there were no differences between the three groups in visual acuity, visual fields and contrast scores. However, there were some differences between the groups despite randomisation, for example, compared to the control and HS groups, the HS + HE group had fewer women (47 % versus 75 % and 75 %) and took more psychotropic drugs (18 % versus 0 % and 0 %).

**Table 1:** Demographic and health-related characteristics of study participants at entry to the trial

Values are numbers (%) unless stated otherwise	Control (*n* = 16)	Home safety only (*n* = 16)	Home safety + Home exercise (*n* = 17)
Mean (SD) age (years)	80.8 (6.9)	81.4 (7.5)	82.1 (8.7)
Women (%)	12 (75)	12 (75)	8 (47)
Ethnicity^a^ (%)			
White-British	16 (100.0)	14 (87.5)	16 (94.1)
White-Irish	0 (0.0)	1 (6.2)	0 (0.0)
White-Other	0 (0.0)	1 (6.2)	1 (5.9)
One or more fall(s) in previous 6 months (%)	8 (50)	6 (38)	1 (6)
Annualised^b^ fall rate per person at baseline by retrospective recall method (95 % CIs)	1.50 [0.78,2.62]	1.63 [0.87,2.78]	0.47 [0.13,1.20]
Mean (SD) number of medications	3.56 (2.42)	4.69 (3.00)	3.35 (2.18)
Takes psychotropic medication? (yes/no)	0	0	3 (18)
Lives alone	9 (56)	7 (44)	9 (53)
Does not follow home safety measures at baseline	7 (44)	7 (44)	7 (41)
Has not attended classes to improve fitness	11 (69)	10 (63)	12 (71)
Walks outside on own regularly	9 (56)	9 (56)	8 (47)
Uses walking aid to walk outside on own	4 (25)	7 (44)	8 (47)
Sight loss registered? (%)			
No	6 (38)	7 (44)	1 (6)
Impaired	4 (24)	5 (31)	5 (29)
Severe	6 (38)	4 (25)	11 (65)
Duration of visual impairment in years (%)			
0–6	3 (19)	2 (13)	2 (12)
7–10	6 (38)	6 (38)	3 (17)
11–20	2 (13)	5 (31)	5 (30)
21–59	1 (6)	1 (6)	2 (12)
60–92	0	1 (6)	2 (12)
Missing	4 (24)	1 (6)	3 (17)
Baseline vision scores			
Visual acuity			
Mean (SD) logMAR scores	1.17 (0.31)	1.05 (0.37)	1.15 (0.29)
Right eye/left eye	1.07 (0.37)	1.05 (0.35)	1.10 (0.33)
Visual fields			
Mean (SD) scores	72.14 (28.11)	75.90 (22.12)	64.50 (28.73)
Esterman test	*n* = 7 (44 %)	*n* = 10 (63 %)	*n* = 10 (59 %)
Contrast sensitivity tests			
Mean (SD) scores			
(1) Pelli-Robson	0.77 (0.38) *n* = 7	0.95 (0.40) *n* = 10	0.75 (0.44) *n* = 10
(2) MARS	0.95 (0.93) *n* = 9	0.77 (0.30) *n* = 6	0.71 (0.47) *n* = 7
Eye conditions (%)			
Age-related macular degeneration	8 (50)	12 (75)	8 (46)
Glaucoma	3 (19)	2 (13)	2 (12)
Myopic degeneration	2 (12)	0	1 (6)
Diabetic retinopathy	0	1 (6)	0
Other	0	0	3 (18)
Missing	3 (19)	1 (6)	3 (18)

^a^Self-reported; ^b^Exact Poisson rate

### Response rate and follow-up of primary and secondary outcome measures

#### Primary outcome

The majority of participants completed and returned the falls diaries (41/49, 83.7 %) and exercise calendars (15/17, 88.2 %). Although, only one person was identified as unable to complete the diaries, in practice, varying numbers of patients each month did not complete and return the diaries because, for example, they forgot, or did not have anyone to take it to the postbox. When diaries were not returned, individuals were telephoned, and thus data was obtained from 43 study participants over 6 months’ follow-up.

#### Secondary outcomes

Participants appeared to understand and complete Phone-FITT, SF-12, VCM1, and Short FES-I appropriately. AFRIS was unsuitable for the control group because the questions related to home safety modifications and exercise interventions, which they had not received.

### Parameters for main study

It should be recalled that this feasibility study was not powered to detect statistical differences and, thus, it is no surprise that we did not detect any statistical differences between the three groups in the number of falls at 6 months (13 in control, 19 in HS only, 18 in HS + HE groups, respectively, *p* = 0.8, Table 2). There were no statistically significant differences between the groups in the number of injurious falls (*p* = 0.4). The difference in annualised rate of falls per group was also not statistically significant; control 1.58 (95 % CI = 0.84 to 2.71), HS 2.32 (1.40 to 3.62) and HS + HE 2.22 (1.31 to 3.50). Sample size calculations for a full trial were undertaken based on a primary outcome of an annualised rate [9] and found that 184 in each group (HS and HS + HE) would have 80 % power to detect a difference between annualised fall rates of 1.17 and 1.65 (i.e. 29.1 % reduction in falls assuming complete data and a shape parameter of 0.643 estimated from our data), using a negative binomial regression model with a 5 % two-sided significance level [44].

**Table 2:** Outcome measures: fall events and injurious falls; fear and attitudes to falling; and quality of life measures

	Control (*n* = 13)	Home safety only (*n* = 15)	Home safety + Home exercise (*n* = 15)
Number of falls over 6 months	13	19	18
Annualised^a^ fall rate per person over follow-up period by self-report fall calendar method (95 % CIs)	1.58 (0.84–2.71)	2.32 (1.40–3.62)	2.22 (1.31–3.50)
Number (% of group) with ≥1 fall(s)	8 (50)	7 (44)	9 (53)
Number (% of group with ≥2 falls	3 (19)	5 (31)	3 (18)
Injurious falls – severity of injury (%)			
Serious injury	0	2 (10)	0
Moderate injury	5 (38)	7 (37)	6 (33)
Minor injury	1 (8)	4 (21)	3 (17)
No injury	7 (54)	6 (32)	9 (50)
Injurious falls per person per year			
Serious	0 (0–0.45)	0.24 (0.03–0.88)	0.00 (0–0.45)
Moderate	0.24 (0.03–0.88)	0.85 (0.34–1.76)	0.37 (0.08–1.08)
AFRIS Mean (SD)			
Baseline: strength and balance training	NA	33.38 (5.60)	32.06 (6.81)
6-month follow-up: strength and balance training	NA	30.33 (6.90)	32.64 (4.97)
Baseline: home safety improvements	NA	33.69 (5.76)	34.18 (3.94)
6-month follow-up: home safety improvements	NA	32.33 (6.51)	33.14 (5.22)
Short FES-I Mean (SD)			
Baseline	11.13 (2.92)	11.56 (4.21)	14.41 (6.81)
6-month follow-up	10.38 (2.02)	12.93 (5.64)	11.50 (4.70)
VCM1 Mean (SD)			
Baseline	2.29 (0.10)	2.21 (0.83)	2.19 (1.12)
6-month follow-up	2.50 (0.84)	2.30 (0.75)	2.10 (1.19)
SF-12 Mean (SD)			
Baseline: physical	43.17 (13.47)	41.60 (10.09)	42.89 (10.79)
6-month follow-up: physical	46.03 (11.39)	42.89 (9.10)	43.21 (8.61)
Baseline: mental	48.71 (8.39)	51.41 (8.40)	49.70 (7.61)
6-month follow-up: mental	46.72 (11.49)	48.87 (12.23)	54.35 (6.89)

AFRIS, views about strength/balance and home safety. A higher score indicates an increased agreement about the benefits (range = 6–42)

Short FES-I, this is a ‘fear of falling’ scale which measures concern about falling. A higher score indicates more concern (range = 7–28)

VCM1, measures vision-related quality of life. A higher score suggests more concern about vision. (range = 0–5)

SF-12, measures functional health and wellbeing. A higher score indicates better health (range 0–100)

There were no statistical differences between groups with regard to changes in Short FES-I, VCM1 and SF-12 scores (Table 2).

### Adherence to the interventions

The most common hazards identified during the HS assessment were outside steps and internal flooring. The OT completed a 6-month follow-up telephone call with 30/33 (91 %) of those in the two HS groups. Either full or partial adherence to the HS recommendations across the HS and HS + HE groups was 100 % (HS, *n* = 15, 14 adhered fully and one partially; HS + HE, *n* = 15, two had no recommendations, 11 adhered fully and two partially). Three types of HS interventions were applied equally in both groups: OT actions (e.g. taping steps) averaged 1.1 per participant in the HS group and 0.67 in the HS + HE group; recommendations for which participants were responsible (e.g. to improve lighting) averaged 3.1 and 3.47, respectively; and referrals to other services at 0.67 and 0.87, respectively.

**Fig. 2:**
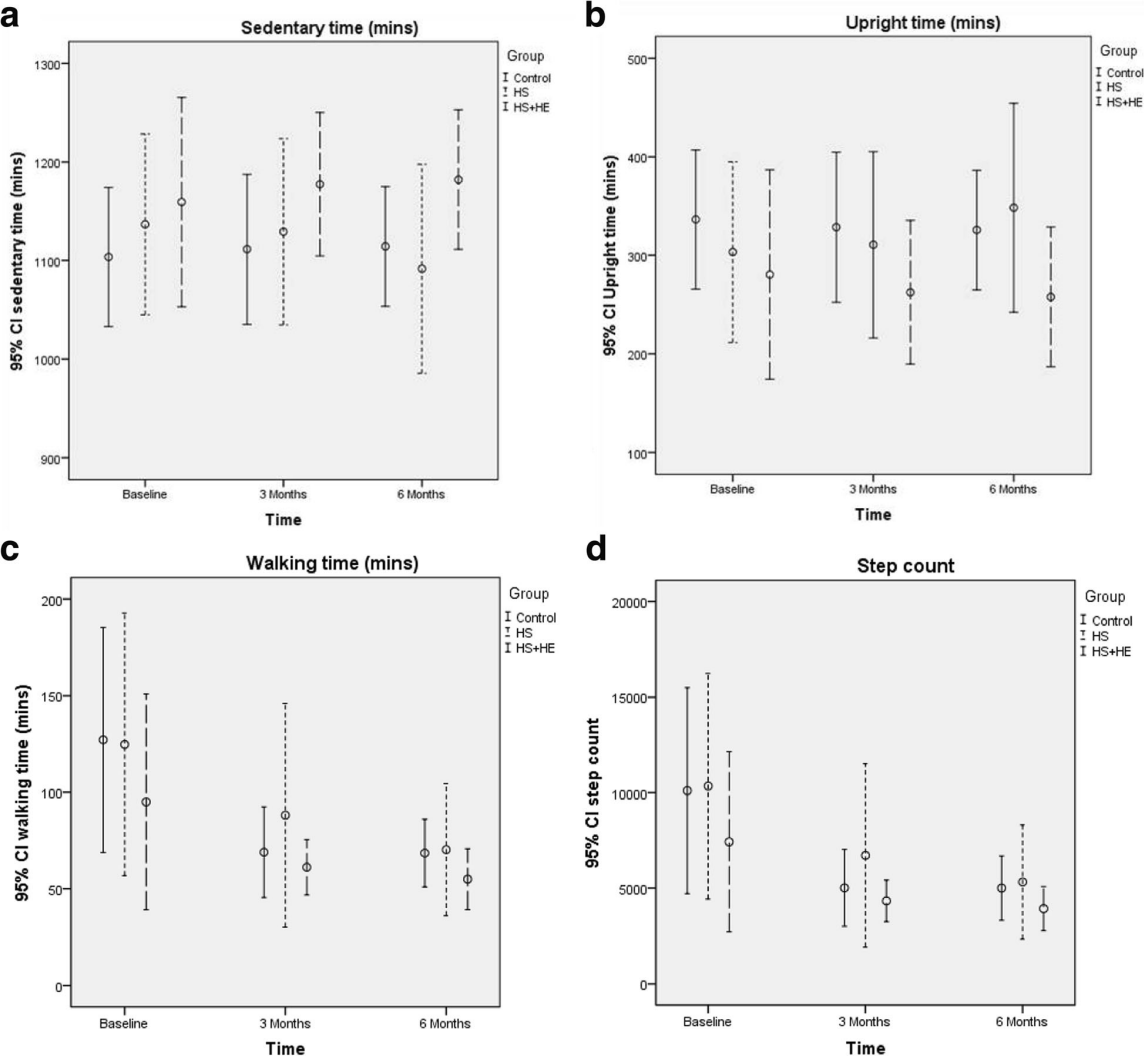
Activity data recorded by activPAL monitors at baseline and 3- and 6-month follow-ups

Adherence to exercise (or, in the case of the control group, exercise levels without an intervention) was measured using exercise calendars, Phone-FITT (self-report) and activPAL (instrumented) approaches. There was disagreement between self-report and instrumented exercise measurements. Whilst there were changes from baseline in exercise, there were no significant differences between groups at 6 months and the group-by-time interactions were also not significant (Table 3). In the HS + HE group during the follow-up period, 86.7 % of participants reported in their exercise diaries that they exercised on average three times a week (Table 3). Mean recreational summary scores for frequency, duration and intensity (TSS_FDI) on the self-report Phone-FITT increased significantly over time in all three groups (Table 3), although there were no significant group or group-by-time differences. The instrumented activPAL data revealed a significant (*p* < 0.001) reduction in both walking time and step count between baseline and subsequent months (Table 3, Fig. 2). A log transformation was required for walking time, for the model diagnostics to be satisfied. A sensitivity analysis using linear mixed models reached the same conclusions, and these test results are consistent with the size of the confidence intervals observed in Fig. 2 panels a-d.

**Table 3:** Adherence to exercise as measured by exercise calendars (self-report), Phone-FITT (self-report) and activPAL (activity monitors)

Mean (SD)	Control (*n* = 13)	Home safety only (*n* = 15)	Home safety + Home exercise (*n* = 15)
Exercise calendar	NA	NA	86.7 % (13/15)
Phone-FITT (TSS_FDI)			
Baseline	36.86 (15.16)	35.23 (8.04)	31.66 (13.16)
3-month	47.93 (14.94)	35.63 (11.30)	42.56 (13.62)
6-month	51.05 (25.72)	47.37 (18.64)	51.97 (22.27)
GLM tests^c^ for (i) time *p* < 0.001, (ii) group-by-time *p* = 0.2, (iii) group *p* = 0.3			
activPAL^a^ mean (SD) [median]			
Walking time (min)			
Baseline	127.1 (81.4) [96]	124.7 (101.2) [120]	95.0 (78.1) [69]
6-month follow-up	68.5 (22.9) [71]	70.2 (44.5) [59]	55.0 (24.8) [50]
GLM tests^b,c^ for (i) time *p* < 0.001, (i) group-by-time *p* = 0.8, (iii) group *p* = 0.4			
Step count			
Baseline	10,103 (7542) [6254]	10,339 (8797) [8956]	7426 (6588) [5110]
6-month follow-up	5000 (2192) [4962]	5321 (3892) [4128]	3927 (1815) [3446]
GLM tests^b,d^ for (i) time *p* < 0.001, (ii) group-by-time *p* = 0.8, (iii) group *p* = 0.5			

It also measures these in relation to frequency, duration and intensity. Scores are summed to give a total. A higher score suggests a higher activity rate.

^a^Median also presented for skewed data

^b^Based on natural log data at three time-points

^c^Sphericity assumed

^d^Greenhouse-Geisser statistic Phone-FITT, measures physical activity in two main areas; recreational and household

*GLM* general linear model

### Economic evaluation

The resources and costs of delivering the interventions are shown in Table 4 and it is noteworthy that there is considerable intragroup variability on a number of resource and cost items. The average (per patient) cost of delivering the interventions was £249 in the HS group and £674 in the HS + HE group over the 6 months, including full economic costing of the PMs. The primary outcome analysis showed no significant difference in the number of falls between groups so a cost per fall prevented by the intervention was not calculated.

**Table 4:** Resource utilisation and costs of the interventions (pounds sterling, 2011)

		Home safety only (*n* = 15)	Home safety + Home exercise (*n* = 15)	
		OT	OT	Peer mentor
		Mean (SD)	Mean (SD)	Mean (SD)
Telephone calls	Number	3.7 (1.5)	4.7 (2.2)	3.1 (3.3)
	Min	13.9 (6.7)	21.5 (11.6)	29.5 (25.4)
	Cost (£)	19.1 (9.2)	29.5 (16.0)	13.3 (11.4)
Home visits	Number	2.3 (0.5)	3.1 (0.6)	2.5 (0.5)
	Min	110.0 (38.5)	210.7 (82.3)	175.9 (55.6)
	Cost (£)	150.7 (52.7)	288.6 (112.7)	135.4 (42.9)
Patient-related non-face-to-face	Travel (min)	21.7 (7.8)	23.9 (7.3)	144.1 (52.9)
	Notes etc. (min)	71.3 (34.9)	115.6 (29.2)	Not applicable
	Cost (£)	65.1 (23.5)	97.7 (21.5)	64.8 (23.8)
Travel costs	Cost (£)	13.82 (6.7)	23.5 (13.4)	20.9 (10.8)
Total average costs (£)		Mean £248.71	Mean £674.32	

Community OT, £82 per hour patient contact, i.e. visits and telephone calls; £42 per hour patient-related non-face-to-face; peer mentor based on family support worker, £46 per hour client-related work, i.e. visits and telephone calls; £27 per hour patient-related non-face-to-face [43]. Travel costs at 45p per mile

### Acceptability of the interventions

Participants varied in their reaction to the HS assessment. About half suggested that modifications were seen as common sense and were welcomed. A few had initial doubts about what was suggested by the OT, but later agreed that the adaptation had been helpful, having allowed the changes to be made.

From analysis of the interviews a number of themes were identified related to acceptability, many of which resonate with other qualitative work in the area [45, 46]. The main barrier to full engagement with HS was resistance to change, which was subdivided into (1) not believing that an adaptation was necessary, (2) reluctance to remove familiar possessions, and (3) perceived threats to independence. A small number of older people resisted changes on aesthetic grounds such as disliking the look of some adaptations or choosing to keep a hazardous rug because they preferred the look of the room with it:

‘I think she did suggest I put a handrail down to the garden, which I haven’t done yet, and I don’t really think, if I’m careful, I need.’ P049 (female, aged 96)

People who reportedly already exercised or believed that the exercises would be beneficial to them easily engaged with the HE programme. Having a programme created and amended personally for them by the OT helped participants to keep on exercising. Encouragement from family members, the OT and PMs increased acceptability:

‘It was encouragement. It was nice to chat to somebody about how I was going on. It was nice to have it confirmed that I was actually doing the right thing along the right track, and yes, it did help in that way.’ P009 (male, aged 76)

About a third of the older people reportedly looked forward to doing the exercises and enjoyed having something to focus on. Noticing positive results helped people stick with the programme and a few relied on the belief that the exercises would be good for them in the long run:

‘I find I can walk now. At one point, if I set off to walk to the bowling green in the park, it’s only, what 300 yards, after 100 yards, my ankles were screaming at me, aching you know. Well now, I can walk right to there no trouble.’ P021 (male, aged 83)

Another third of participants thought the programme might be too difficult or would not be beneficial for them. Those with active lives often felt that they had something better to be doing with their time, and so did not commit to the programme.

Discomfort during or after exercising and severe impairments meant the programme was too burdensome for a few participants:

‘I couldn’t follow them … I had the greatest difficulty in trying to do it, and they pack so much into one time that by the time you’ve finished you’ve forgot how to do the first one … I can’t see any damned thing … I can’t do it!’ P020 (male, aged 97)

The OT’s approach was vital in securing engagement with both interventions. The OT was remembered positively by all intervention participants and was described as very pleasant, patient, respectful and knowledgeable. Participants praised how the OT clearly introduced and explained the interventions, and how persuasive she was, without being pushy.

## Discussion

We know that older people with SI have a high risk of falling. We also know that group and individual exercise programmes and home safety assessments and modifications prevent falls in the community-dwelling older population. However, little is known about whether these types of interventions are suitable for older people with SI [8]. Research carried out in New Zealand shows that HS modification rather than HE may be more effective in preventing falls in older people with SI [9]. Using the MRC Framework/Guidance, we have carried out a feasibility study to assess whether it is possible to conduct a full trial in the UK similar to (and learning from) the New Zealand study [12, 13]. We have demonstrated that it is feasible to deliver the interventions, at reasonable cost, to older people with SI, and to conduct a RCT comparing the approaches. We have identified outcome measures and estimated that we will need 184 in each group to complete the study for it to be sufficiently powered on the primary outcome of falls over 12 months. Our findings show that an OT can deliver both HS and HE interventions to ensure consistent advice is provided to participants and this bodes well for wider application in the community. Where there are differences in the health care system compared to that of our (UK) and the original study (New Zealand) then implementation will be effected and, hence, needs to be tested in differing localities. We have also revealed a number of problems for any definitive study.

### Strengthening the HS and HE interventions

We customised a standard HS intervention for older people to be suitable for those with SI that enabled them to be adherent. However, tailoring the OEP to the needs of older people with SI did not result in more physical activity. According to the activPAL instrumented monitoring data, activity levels dropped, although the Phone-FITT questionnaire on physical activity indicated an increase. This could mean:

1. Our feasibility trial appears to have negatively modified participant activity behaviour as measured by activPAL. This would be line with findings of another study which followed up patients after hospital and community-based exercise programmes and found that after the intervention some participants restricted activity to prevent falls [47]
2. It is possible that patients, when responding to the self-report data, may have felt obliged to say what they thought researchers wanted to find leading to contradictory findings
3. It is also possible that many patients in the HE group felt that as they were doing extra strength and balance exercise that they did not need to do so much other walking or physical activity and so they compensated by doing less walking [47]. Alternatively, they may still have perceived themselves to be more active as they were doing the OEP exercises. This would be in keeping with their questionnaire responses as they stated they had exercised as requested and is supported by the activPAL data showing no change in sedentary time despite a reduction in number of steps (Fig. 2)
4. Finally, it is possible that participants changed their walking technique such that the activPAL monitor was no longer always able to identify steps. This is possible and has been seen before in terms of potential inaccuracy of identifying steps with monitors when (e.g.) a shuffling gait is used [48]. However, as sedentary time (which is posture based and more accurate) did not alter this seems unlikely

Given the variety of responses from the qualitative interviews it is likely that that there were different and several behavioural responses within and across patients to the questionanire and instrumented measures of activity in our study. Further work is needed to unpick the differences between the self-report activity data and the activPAL data. In addition, therefore, to subjective measures of activity, an instrumented activity monitor should be used in a full trial and consideration given to whether it is used in all groups.

The implementation of the HS recommendations required one-off input from participants. Adherence to the HE programme, however, requires physical effort on a continuing basis and the ability to read instructions/use an audio compact disc (CD) which could be difficult for people with SI, some of whom lived alone. This suggests that weekly home visits from an exercise trainer may be more successful at improving adherence in this group [11]. Alternatively, involving them in group exercise with additional requisite support so as to facilitate involvement despite their SI, may provide a better progression of strength intensity and challenge in balance. To ensure sufficient dose of exercise the full trial should deliver the HE programme for a 12-month period. Setting specific short-term exercise targets and providing feedback using the sensor data may also help them to be motivated to continue exercising. However, further research is required to test these ideas, including the cost-effectiveness of increased professional input.

### Optimising the full trial

Another difficulty encountered in this feasibility study was with recruiting a sufficient number of participants fitting the inclusion criteria (Fig. 1). We used the same definition for sight impairment as the New Zealand study. Arguably, if we had set it at less than 6/18 Snellen, which is generally regarded by most authorities as sight impaired, we may have had a slightly bigger sample size. However, the problem of recruitment, whether the definition for sight impairment is set at less than 6/18 or less than 6/24 Snellen, may easily be resolved by accessing the Local Authority Register of people who are SI and by using more than one low vision clinic. Alternately, given the large proportion of patients attending the low vision clinic (who did not fulfil our sight impairment criteria), the inclusion criteria could be relaxed to include attendance at the low vision clinic, thus making a full RCT applicable to a larger population. However, the assumption of our study is that SI at a registerable level poses particular problems in both exercising and maintaining a safe environment [9]. Indeed, in the New Zealand study, rates of adherence to HE were noted to be lower in this group than in their previous studies of the older community living population without SI [9]. This means that if the sample is indeed expanded to include those visiting the low vision clinic but not sufficient to reach the definition for SI, then this changes the study altogether and will not actually answer the research question we originally set, especially because the home safety assessment and exercise programme modifications for those with SI may not be applicable for the majority of participants as they can see well enough.

## Conclusion

Useful information was obtained with regards to adherence to interventions and the feasibility of a large RCT to test the effects of HS assessment and modifications, and a HE programme in preventing falls in older people with SI. The study was not powered to find differences in falls between the three groups. Although, the HS intervention appears feasible, further work is required to investigate how best to modify the delivery of the HE intervention to promote adherence, particularly, intensity and progression of the exercise, for older people with SI and an effective way of measuring changes in activity over time. This needs to be completed before a definitive multicentre RCT is implemented.

## Abbreviations

CD: Compact disc
HE: Home exercise programme
HS: Home safety
IRR: Incident rate ratio
NHS: National Health Service, UK
NIHR: National Institute for Health Research, UK
PM: Peer mentor
OEP: Otago Exercise Programme
OT: Occupational therapist
RCT: Randomised controlled trial
SI: Sight impairment

## References

[1] Rubenstein LZ (2006). Falls in older people: epidemiology, risk factors and strategies for prevention. Age Ageing.

[2] Dhital A, Pey T, Stanford MR (2010). Visual loss and falls: a review. Eye.

[3] Hartholt KA, van Beeeck E, Polinder S, van der Velde N, van Lieshout EM, Panneman MJ, van der Cammen TJ, Patka P (2011). Societal consequences of falls in the older population: injuries, health care costs and long term reduced quality of life. J Trauma.

[4] Stewart J, McVittie C (2011). Living with falls: housebound older people’s experiences of health and community care. Eur J Ageing.

[5] Campbell S (2005). Deteriorating vision, falls and older people.

[6] NICE. Falls: assessment and prevention of falls in older people. NICE clinical guideline161. 2013. https://www.nice.org.uk/Guidance/CG161. Accessed 1 May 2014.

[7] Yang Tian Y, Thompson J, Buck D, Sonola L. Exploring the system-wide costs of falls in older people in Torbay. Kings Fund. 2013. http://www.kingsfund.org.uk/sites/files/kf/field/field_publication_file/exploring-system-wide-costs-of-falls-in-torbay-kingsfund-aug13.pdf. Accessed 1 May 2014

[8] Gillespie LD, Robertson MC, Gillespie WJ, Sherrington C, Gates S, Clemson LM, Lamb SE (2012). Interventions for preventing falls in older people living in the community. Cochrane Database Syst Rev.

[9] Campbell AJ, Robertson MC, Grow SJL, Kerse NM, Sanderson GF, Jacobs RJ, Sharp DM, Hale LA (2005). Randomised controlled trial of prevention of falls in people aged > or =75 with severe visual impairment: the VIP trial. BMJ.

[10] Gleeson M, Sherrington C, Lo S, Keay L (2015). Can the Alexander Technique improve balance and mobility in older adults with visual impairments? A randomized controlled trial. Clin Rehabil.

[11] Stevens Z, Carpenter H, Gawler S, Belcher C, Haworth D, Kendrick D, Morris R, Masud T, Skelton DA, Iliffe S (2013). Lessons learnt during a complex, multicentre cluster randomised controlled trial: the ProAct65+ trial. Trials.

[12] MRC. A framework for development and evaluation of RCTs for complex interventions to improve health. 2000. http://www.mrc.ac.uk/documents/pdf/rcts-for-complex-interventions-to-improve-health/. Accessed 16 Nov 2014.

[13] MRC. Developing and evaluating complex interventions: new guidance. 2008. http://www.mrc.ac.uk/documents/pdf/complex-interventions-guidance/. Accessed 16 Nov 2014.

[14] Arain M, Campbell MJ, Cooper CL, Lancaster GA (2010). What is a pilot or feasibility study? A review of current practice and editorial policy. BMC Med Res Method.

[15] NIHR. Central Commissioning Facility. 2013. http://www.nihr.ac.uk/CCF/RfPB/FAQs/Feasibility_and_pilot_studies.pdf. Accessed 1 May 2014.

[16] Brundle C, Waterman H, Ballinger C, Olleveant N, Skelton DA, Stanford P, Todd C. The causes of falls: views of older people with sight impairment. Health Expect. 2015;17: doi:10.1111/hex.12355 10.1111/hex.12355 PMC4949546 25736829

[17] Hodkinson HM (1972). Evaluation of a mental test score for assessment of mental impairment in the elderly. Age Ageing.

[18] Browne RH (1995). On the use of a pilot sample for sample size determination. Stat Med.

[19] Clemson L (1997). Home fall hazards: a guide to identifying fall hazards in the homes of elderly people and an accompaniment to the assessment tool, the Westmead Home Safety Assessment.

[20] La Grow SJ, Robertson MC, Campbell AJ, Clarke GA, Kerse NM (2006). Reducing hazard related falls in people 75 years and older with significant visual impairment: how did a successful program work?. Inj Prev.

[21] Canadian Association of Occupational Therapists (1997). Enabling occupation: an occupational therapy perspective.

[22] Gardner MM, Buchner DM, Robertson MC, Campbell AJ (2001). Practical implementation of an exercise-based falls prevention programme. Age Ageing.

[23] Laventure RME, Dinan SM, Skelton DA (2008). Someone like me: increasing participation in physical activity among seniors with senior peer health motivators. J Aging Phys Act.

[24] U3A. 2014. http://www.u3a.org.uk/. Accessed 16 Nov 2014.

[25] INVOLVE. Payment for involvement: a guide for making payments to members of the public actively involved in NHS, public health and social care research. 2012. http://www.invo.org.uk/wp-content/uploads/2012/11/INVOLVEPayment-Guiderev2012.pdf. Accessed 16 Nov 2014.

[26] Lamb SE, Jørstad-Stein EC, Hauer K, Becker C, ProFaNE group (2005). Development of a common outcome data set for fall injury prevention trials: the Prevention of Falls Network. Europe consensus. J Am Geriatr Soc.

[27] Hauer K, Lamb SE, Jørstad EC, Todd C, Becker C, ProFaNE group (2006). Systematic review of definitions and methods of measuring in randomized controlled fall prevention trials. Age Ageing.

[28] Schwenk M, Lauenroth A, Stock C, Moreno RR, Oster P, McHugh G, Todd C, Hauer K (2012). Definitions and methods of measuring and reporting on injurious falls in randomised controlled fall prevention trials: a systematic review. BMC Med Res Method.

[29] Gill DP, Jones GR, Zou GY, Speechley M (2008). The Phone-FITT: a brief physical activity interview for older adults. J Aging Phys Act.

[30] Chastin SF, Granat MH (2010). Methods for objective measure, quantification and analysis of sedentary behaviour and inactivity. Gait Posture.

[31] Grant PM, Dall PM, Mitchell SL, Granat MH (2008). Activity-monitor accuracy in measuring step number and cadence in community-dwelling older adults. J Aging Phys Act.

[32] Ware JE, Kosinsiki M, Keller SD (1996). A 12-item short-form health survey: construction of scales and preliminary tests of reliability and validity. Med Care.

[33] Frost NA, Sparrow JM, Hopper CD, Peters TJ (2001). Reliability of the VCM1 Questionnaire when administered by post and by telephone. Ophthalmic Epidemiol.

[34] Yardley L, Donovan-Hall M, Francis K, Todd C (2007). Attitudes and beliefs that predict older people’s intention to undertake strength and balance training. J Gerontol Psychol Sci.

[35] Yardley L, Beyer N, Hauer K, Kempen G, Piot-Ziegler C, Todd C (2005). Development and initial validation of the Falls Efficacy Scale International (FES-I). Age Ageing.

[36] Kempen GIJM, Yardley L, van Haastregt JCM, Zijlstra RGA, Beyer N, Hauer K, Todd C (2008). The Short FES-I: a shortened version of the falls efficacy scale-international to assess fear of falling. Age Ageing.

[37] SPSS 20 IBM Corp. IBM SPSS Statistics for Windows, Version 20.0. 2011. http://www-01.ibm.com/support/docview.wss?uid=swg21476197. Accessed 1 May 2014.

[38] Lancaster GA, Dodd S, Williamson PR (2004). Design and analysis of pilot studies: recommendations for good practice. J Eval Clin Pract.

[39] StatsDirect Ltd. StatsDirect statistical software. 2013. http://www.statsdirect.com. Accessed 1 May 2014

[40] Ritchie J, Spencer L, O’Connor W, Ritchie J, Lewis J (2005). Carrying out qualitative analysis. Qualitative research practice.

[41] Ritchie J, Spencer E, Bryman A, Burgess RG (1994). Qualitative data analysis for Applied Policy Research. Analyzing qualitative data.

[42] NVivo 9. 2011. http://download.qsrinternational.com/Document/NVivo9/NVivo9-Getting-Started-Guide.pdf. Accessed 22 Dec 2014.

[43] Curtis L. Unit costs of health and social care. 2011. http://www.pssru.ac.uk/project-pages/unit-costs/2011/index.php. Accessed 1 May 2014

[44] Keene ON, Jones MRK, Lane PW, Anderson J (2007). Analysis of exacerbation rates in asthma and chronic obstructive pulmonary disease: example from the TRISTAN study. Pharm Stat.

[45] Yardley L, Donovan-Hall M, Francis K, Todd C (2006). Older people’s views of advice about falls prevention: a qualitative study. Health Educ Res.

[46] Horne M, Skelton D, Speed S, Todd C. Perceived barriers to initiating and maintaining physical activity among South Asian and White British adults in their 60s living in the United Kingdom: a qualitative study. Ethn Health. 2013;doi:10.1080/13557858.2013.81476238 10.1080/13557858.2013.814762 23834070

[47] Laybourne AH, Biggs S, Martin FC (2011). Predicting habitual physical activity using coping strategies in older fallers engaged in falls-prevention exercise. J Aging Phys Act.

[48] Godfrey A, Culhane KM, Lyons GM (2007). Comparison of the performance of the activPAL professional physical activity logger to a discrete accelerometer-based activity monitor. Med Eng Phys.

